# A Reliability-Generalization Study of Journal Peer Reviews: A Multilevel Meta-Analysis of Inter-Rater Reliability and Its Determinants

**DOI:** 10.1371/journal.pone.0014331

**Published:** 2010-12-14

**Authors:** Lutz Bornmann, Rüdiger Mutz, Hans-Dieter Daniel

**Affiliations:** 1 Max Planck Society, Munich, Germany; 2 Professorship for Social Psychology and Research on Higher Education, ETH Zurich, Zurich, Switzerland; 3 Evaluation Office, University of Zurich, Zurich, Switzerland; University of Glasgow, United Kingdom

## Abstract

**Background:**

This paper presents the first meta-analysis for the inter-rater reliability (IRR) of journal peer reviews. IRR is defined as the extent to which two or more independent reviews of the same scientific document agree.

**Methodology/Principal Findings:**

Altogether, 70 reliability coefficients (Cohen's Kappa, intra-class correlation [ICC], and Pearson product-moment correlation [r]) from 48 studies were taken into account in the meta-analysis. The studies were based on a total of 19,443 manuscripts; on average, each study had a sample size of 311 manuscripts (minimum: 28, maximum: 1983). The results of the meta-analysis confirmed the findings of the narrative literature reviews published to date: The level of IRR (mean ICC/r^2^ = .34, mean Cohen's Kappa = .17) was low. To explain the study-to-study variation of the IRR coefficients, meta-regression analyses were calculated using seven covariates. Two covariates that emerged in the meta-regression analyses as statistically significant to gain an approximate homogeneity of the intra-class correlations indicated that, firstly, the more manuscripts that a study is based on, the smaller the reported IRR coefficients are. Secondly, if the information of the rating system for reviewers was reported in a study, then this was associated with a smaller IRR coefficient than if the information was not conveyed.

**Conclusions/Significance:**

Studies that report a high level of IRR are to be considered less credible than those with a low level of IRR. According to our meta-analysis the IRR of peer assessments is quite limited and needs improvement (e.g., reader system).

## Introduction

Science rests on journal peer review [1]. As stated in a British Academy report, “the essential principle of peer review is simple to state: it is that judgements about the worth or value of a piece of research should be made by those with demonstrated competence to make such a judgement … With publications, an author submits a paper to a journal … and peers are asked to offer a judgement as to whether it should be published. A decision is then taken, in the light of peer review, on publication” [2] (p. 2). Quality control undertaken by peers in the traditional peer review of manuscripts for scientific journals is an essential part in most scientific disciplines to reach valid and reliable knowledge [3].

According to Marsh, Bond, and Jayasinghe [4], the most important weakness of the peer review process is that the ratings given to the same submission by different reviewers typically differ. This results in a lack of inter-rater reliability (IRR). Cicchetti [5] defines IRR as “the extent to which two or more independent reviews of the same scientific document agree” (p. 120). All overviews of the literature on the reliability of peer reviews published so far come to the same conclusion: There is a low level of IRR [5], [6], [7], [8]. However, these reviews describe the existing literature using the narrative technique, without attempting any quantitative synthesis of study results. From the viewpoint of quantitative social scientists, narrative reviews are not very precise in their descriptions of study results [9]. The term meta-analysis refers to “the statistical analysis of a large collection of analytical results from individual studies for the purpose of integrating the findings” [10] (p. 3). Marsh, Jayasinghe, and Bond [11] note the relevance of meta-analysis to synthesizing results of peer review research. In peer review research, previously published meta-analyses investigated only gender differences in the selection process of grant proposals [12], [13].

In this study, we test whether the result of the narrative techniques used in the reviews – that there is a generally low level of IRR in peer reviews – can be confirmed using the quantitative technique of meta-analysis. Additionally, we examine how the study-to-study variation of the reported reliability coefficients can be explained by covariates. What are the determinants of a high or low level of IRR [7]?

## Materials and Methods

### Literature Search

We performed a systematic search of publications of all document types (journal articles, monographs, collected works, etc.). In a first step, we located several studies that investigated the reliability of journal peer reviews using the reference lists provided by narrative overviews of research on this topic [5], [6], [7], [8] and using tables of contents of special issues of journals publishing research papers on journal peer review (e.g., *Journal of the American Medical Association*). In a second step, to obtain keywords for searching computerized databases, we prepared a bibliogram [14] for the studies located in the first step. The bibliogram ranks by frequency the words included in the abstracts of the studies located. Words at the top of the ranking list (e.g., peer review, reliability, and agreement) were used for searches in computerized literature databases (e.g., Web of Science, Scopus, IngentaConnect, PubMed, PsycINFO, ERIC) and Internet search engines (e.g., Google). In a third step of our literature search, we located all of the citing publications for a series of articles (found in the first and second steps) for which there are a fairly large number of citations in Web of Science.

The search for publications identified 84 studies published between 1966 and 2008. Fifty-two out of the 84 studies reported all information required for a meta-analysis: reliability coefficients and number of manuscripts. Nearly all of the studies provided the following quantitative IRR coefficients: Cohen's Kappa, intra-class correlation (ICC), and Pearson product-moment correlation (r). If different coefficients were reported for the same sample in one single study, ICCs were included in the meta-analyses (n = 35). The ICC measures inter-rater reliability and inter-rater agreement of single reviewers [15]. An ICC is high, if reviewers absolutely agree in their ratings of the same manuscript (absolute consensus) and rate different manuscripts quite differently (consistency). With a high ICC, the average rating of reviewers across all manuscripts in the sample can be accurately inferred from the individual ratings of reviewers for a manuscript. If there were no ICCs available (n = 35), r (n = 9) or Cohen's Kappa (n = 26) was used. Of the 52 studies, 4 could not be included because they reported neither ICC nor r nor Cohen's Kappa. In the end, we had 48 studies [16]–[65] (two studies reported their findings in two papers). As some of the studies reported more than one reliability coefficient for various journals and different cohorts of submissions, we had 70 reliability coefficients for the analyses (on average 1.5 coefficients per study). The studies included were based on a total of 19,443 manuscripts. On average, each study had a sample size of 311 manuscripts; the average sample size per study ranged between 28 and 1983 manuscripts (some studies were based on more than one sample).

### Statistical Procedure

Reliability generalization studies were originally introduced by Vacha-Haase [66] to summarize the score reliabilities across studies while searching for the source of variability in studies' reliabilities. In our study we focus on the inter-rater reliabilities of journal peer reviews instead of score reliabilities. The technique involves pooling together the reported IRR estimates and applying meta-analytic techniques to sum up commonalities and differences across studies [67]. There are two ways to conceptualize this summarization: fixed effects models and random effects models. Following Hedges [68] and Hedges and Vevea [69], the fixed effects model implies that the IRR in the population is assumed to be the same for all studies included in the meta-analysis (homogeneous case). Therefore, the only reason the IRR estimates varies between studies is sampling error, that is, the error in estimating the reliability. The theoretically defined standard error of the IRR coefficient indicates the amount of sampling error. The standard error, however, depends strongly on the sample size: The higher the sample size of a study, the lower the standard error of the reliability coefficient is, and the better the information of this study is for the estimation of the overall true reliability. Therefore, in summing up the reliabilities across studies to a mean value, studies with large samples sizes will be more heavily weighted (1/standard error as weight) than studies with low sample sizes.

As opposed to fixed effects models, the objective of random effects models is not to estimate a fixed reliability coefficient but to estimate the average of a distribution of reliabilities. Random effects models assume that the population effect sizes themselves vary randomly from study to study and that the true inter-rater reliabilities are sampled from a universe of possible reliabilities (“super-population”).

Whereas fixed effects models only allow generalizations about the studies that are included in the meta-analysis, in random effects models the studies are assumed to be a sample of all possible studies that could be done on a given topic, about which the results can be generalized [70]. From a statistical point of view, the main difference between fixed effects and random effects models is in the calculation of standard errors associated with the combined effect size. Fixed effects models only use within-study variability to estimate the standard errors. In random effect models, two sources of error variability are taken into account: within-study variability and between-study variability. Within the framework of random effects models it can be tested whether the between-study variability deviates statistically significant from zero and whether a fixed effects model is sufficient to fit the data, respectively (Q test).

Multilevel models are an improvement over fixed and random effects models, as they allow simultaneous estimation of the overall reliability and the between-study variability and do not assume the independency of the effect sizes or correlations. If a single study reports results from different samples, the results might be more similar than results reported by different studies. Statistically speaking, the different reliability coefficients reported by a single study are not independent. This lack of independence may distort the statistical analyses – particularly the estimation of standard errors [71], because the effective sample size decreases with increasing similarity among the units of analysis (i.e., the samples of a single study). Multilevel models take into account the hierarchical structure of data and are therefore able to deal with the dependence problem by including different samples for a single study as an additional level of analysis. With respect to reliability generalization studies, Beretvas and Pastor [67] suggested a three-level model (which we used in this paper as follows: first level: manuscript, second level: sample, third level: study). Whereas the variability of the reliability coefficients between different samples within single studies (level 2) and the variability between studies (level 3) are estimated by multilevel models, the within-variability (standard error, level 1) must be calculated for each study using the standard error of the reliability coefficient and will be imputed in the multilevel analysis.

In this study we used a multilevel model (especially a three-level model) suggested by several researchers, including DerSimonian and Laird [72], [73], DerSimonian and Kacker [74], Goldstein, Yang, Omar, Turner, and Thompson [75], van den Noortgate and Onghena [76], Beretvas and Pastor [67], and van Houwelingen, Arends, and Stijnen [77].

When there is a high level of between-study variation (study heterogeneity), it is important to look for explanatory variables (covariates) to explain this variation. As Egger, Ebrahim, and Smith [78] argued, “the thorough consideration of heterogeneity between observational study results, in particular of possible sources of confounding and bias, will generally provide more insights than the mechanistic calculation of an overall measure of effect” (p. 3). To explain the study heterogeneity of the inter-rater reliabilities in this study, meta-regression analyses were calculated. Whereas ordinary linear regression uses individual data from a single study, meta-regression uses weighted data from multiple studies, where each study provides for a data point in the regression analysis. To include categorical covariates (e.g., disciplines) in the meta-regression, they were dummy-coded. To avoid an excessive reduction of sample size and to warrant the power of the statistical tests, the missing values in categorical covariates are coded as an additional category, called “unknown.” In total, 32 studies reporting 44 reliability coefficients (ICC or r) could be included in the meta-regression analyses. Following the recommendations of Baker, White, Cappelleri, Kluger, and Coleman [79], we thus had a sufficient number of studies to run a linear meta-regression with two or more covariates.

### Proposed Covariates

The following covariates were included in the meta-regression analysis:

#### (1) Number of manuscripts

The number of manuscripts was used as the first covariate, based on which the reliability coefficients in the individual studies were calculated. The number of manuscripts was divided by 100 to obtain a regression parameter that is not too small. This procedure both warrants the accuracy of estimation and enhances the interpretation of the results. The influence of the commonly called “publication bias” or “file drawer problem” [80] (p. 150) is tested with this covariate: “Publication bias is the tendency on the parts of investigators, reviewers, and editors to submit or accept manuscripts for publication based on the direction or strength of the study findings” [81] (p. 1385). Hopewell, Loudon, Clarke, Oxman, and Dickersin [82] found, e.g., that clinical trials with positive or statistically significant findings are published more often, and more quickly, than trials with negative or statistically not significant findings. It is well known in statistics that even very low correlations or – in our case – IRR coefficients are still statistically significant, if only the sample size of the study is high, et vice versa, high IRR coefficients are statistically significant, even if the sample size of the study is small. Therefore, Hox [80] recommended including the sample sizes of the studies as a covariate in a multilevel meta-analysis.

#### (2) Method

According to the findings of an analysis by Cicchetti and Conn [24], inter-rater reliabilities vary considerably in dependence on the method with which the reliabilities were calculated in the empirical studies. For this reason, the method used for the calculation of the IRR (ICC or r) in a study was considered in the meta-regression analyses as a second covariate. Higher coefficients are to be expected when using the one or other method. Only in the case where ratings by different reviewers have identical means and variances are r and one-way ICC identical [83]. Otherwise, r considerably overestimates the amount of IRR [36]. To include ICC and r into one single analysis, we followed Thompson and Vacha-Haase [84] and used the square root of the ICC as a kind of correlation coefficient. Fisher Z-transformed correlations and the corresponding standard error are used instead of correlations (square root of the reliability), because correlations are not continuous. The Fisher Z-transformation yields an approximate continuous scale.

#### (3) Discipline

As a third covariate the scientific discipline was included in the meta-regression analysis: (1) economics/law, (2) natural sciences, (3) medical sciences, or (4) social sciences. For Weller [7] “some discipline differences were apparent in reviewer agreement studies. Many of the studies were conducted in psychology and sociology and to some degree medicine, where the subject matter is human behavior and human health. These areas are less precise and absolute than other sciences and, therefore, it might be expected that there are more discussions of reviewer agreement” (p. 200).

#### (4) Object of appraisal

The fourth covariate is based on the object of appraisal. According to Weller [7], higher levels of inter-rater reliabilities are to be expected for abstracts that are submitted especially at conferences or meetings than for papers (such as research articles or short communications), which are normally submitted to journals: “Abstracts by their very nature are an abbreviated representation of a study. Reviewers of abstracts are asked to make a recommendation to accept or reject a work with little knowledge of the entire endeavor. One would expect studies of reviewer agreement of abstracts to show a relatively high level of reviewer disagreement” (p. 183).

#### (5) Cohort

Further, with the covariate cohort, the period is included in the meta-regression analyses on which the data in a study is based. In general, a study investigated the IRR for manuscripts submitted to a journal within a certain period of time (e.g., one year). For the meta-analysis, we classified these periods into four different categories of time (e.g., 1980–1989). The covariate cohort tests whether the level of IRR has changed since 1950.

#### (6) Blinding

“In an attempt to eliminate some of the drawbacks of the peer review system, many journals resort to a double-blind review system, keeping the names and affiliations of both authors and referees confidential” [85] (p. 294). In a single-blind system, the reviewer knows the identity of the author but the reviewer remains anonymous. One of the drawbacks meant to be eliminated by use of the double-blind system is the low level of IRR. If the reviewer's ratings are not to be influenced by potential sources of bias (such as the author's gender or affiliation), a higher level of agreement between reviewers is to be expected. We tested the extent to which the type of blinding can actually influence the level of IRR.

#### (7) Rating system

Finally, the type of rating system used by the reviewers in a journal peer review process (analyzed in a reliability study) was included as a covariate. This tests whether various rating systems (metric or categorical) are connected to different levels of IRR. Strayhorn, McDermott, and Tanguay [60] were thus able to determine that reliability increased by increasing the number of rating scale points for questions about a manuscript. In some studies that we included in this study, there were no references to the rating system to be found (coded for the regression analysis as “unknown”). In a narrative review about studies on the reliability of peer review, Cicchetti [5] stated that information about the rating system is very basic for an empirical research paper and criticized studies that did not provide this information. Thus, their mention or non-mention can provide information about the quality of a study.

### Software

All analyses were performed using SAS PROC MIXED in SAS, version 9.1.3 [86]. The SAS syntax suggested by van Houwelingen, Arends, and Stijnen [77] was used.

## Results

### Comparison of Average Effects

Using the above mentioned meta-analysis methods, three analyses were calculated based on r coefficients and ICC coefficients (see Table 1, part a). The different meta-analysis methods estimate mean correlations that were squared again to obtain reliability coefficients as the ICC. A very low average reliability (∼.23) with a 95% confidence interval of ∼.22 to ∼.25 was obtained for the fixed effects model. The results for the random effects model showed a slightly higher average reliability (∼.34) with a 95% confidence interval of ∼.29 to ∼.39. An ICC of .23 indicates that only 23% of the variability in the reviewers' rating of a manuscript could be explained by the agreement of reviewers. The residue of 77% traces back to disagreement among the reviewers' ratings.

**Table 1:** Overview of mean reliabilities with confidence interval.

*Method*	*Publication*	*Levels*	*N*	*Mean*	*CL95%*	*CU95%*
*a) ICC, r*						
Fixed effects	van Houwelingen, Arends and Stijnen [77]	2	44	.234	.222	.246
Random effects	van Houwelingen, Arends and Stijnen [77]	2	44	.341	.289	.392
	van Houwelingen, Arends and Stijnen [77]	3	44	.340	.283	.396
*b) Cohen's Kappa*	Hunter & Schmidt [107], [108], [109]	2	26	.17	.13	.21

**Notes**: To obtain the reliability estimates (ICC/r^2^) shown in this table, correlations (r) were squared. N = number of coefficients included. Levels = number of levels in the meta-analysis.

One further model was calculated on the basis of Cohen's Kappa (see Table 1, part b). The mean reliability amounts to .17. The confidence interval varies between .13 and .21. According to the guidelines for interpretation of Kappa by Landis and Koch [87], these mean reliabilities indicated a slight IRR. A Cohen's Kappa of .17 indicates that the reviewers agreed in their evaluations for 17% more of the manuscripts than would have been predicted on the basis of chance alone [88].

**Figure 1:**
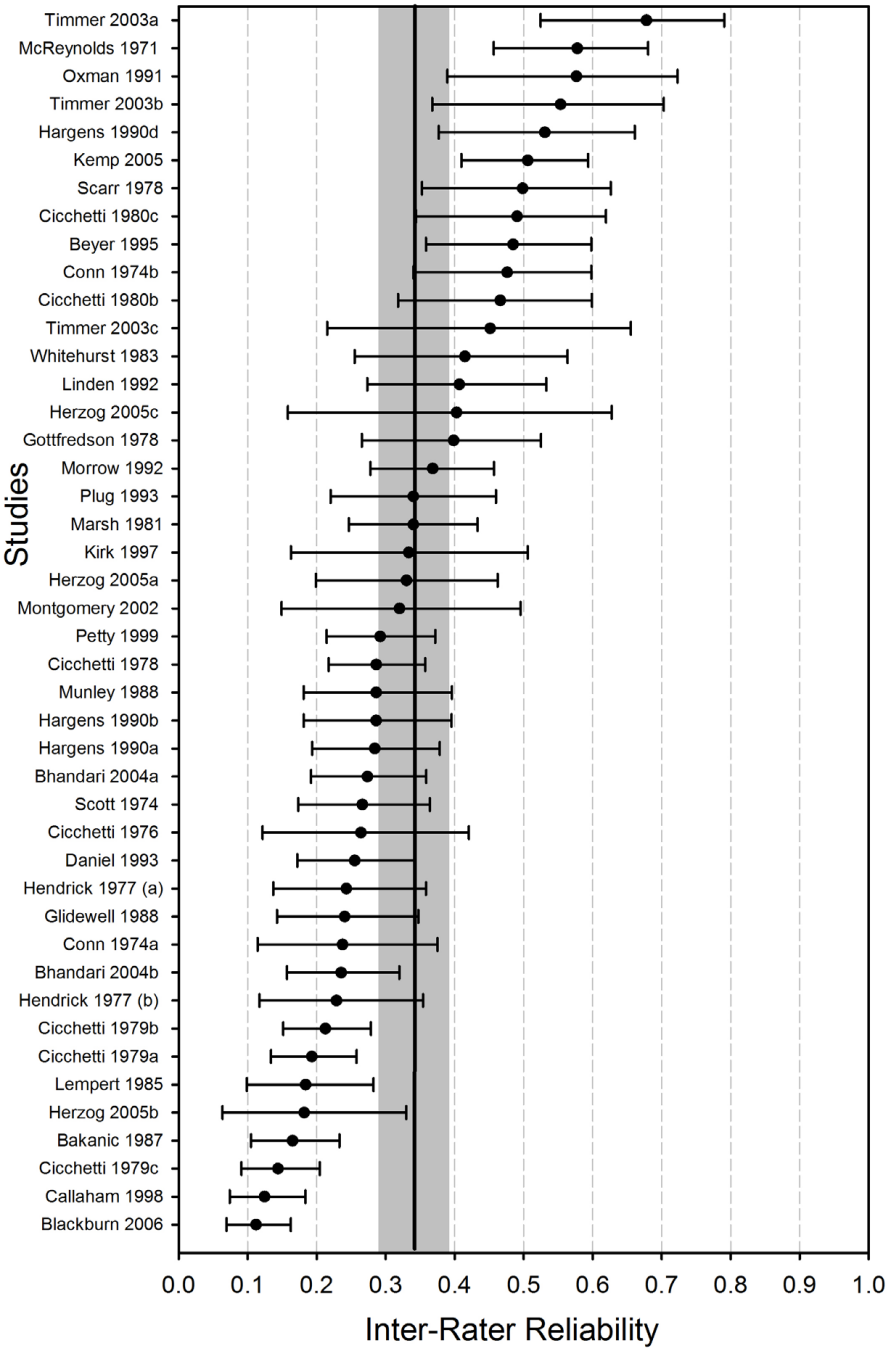
Forest plot of the predicted inter-rater reliability (Bayes estimate) for each study (random effects model without covariates) with 95% confidence interval (as bars) for each reliability coefficient (sorted in ascending order). The 95% confidence interval of the mean value (vertical line) is shaded grey. Predicted values for the same author and year but with different letters (e.g., Herzog 2005a and Herzog 2005b) belong to the same study.

The forest plot (Figure 1) shows the predicted inter-rater reliabilities for each study and the individual 95% confidence interval for each reliability coefficient (r coefficient or ICC coefficient) based on the three-level model [77]. The predicted coefficients are Bayes estimates [80]. Bayes estimates take into account the different sampling errors of the reliability coefficients. The smaller the sampling error of a study and thus the larger its sample size (manuscripts) is, the more the reported reliability coefficient is a true estimate of the reliability of the study. The larger the sampling errors of a study and thus the smaller its sample size, the more the mean value across all reliability coefficients is a true estimate of the reliability of the particular study. This means that the smaller the sample sizes of the studies included in the meta-analysis are, the more the empirical Bayes estimates are shrunken towards the overall mean ß_0_. As Figure 1 shows, there was a positive correlation between the extent of IRR and the individual confidence interval: The smaller the coefficient, the smaller the confidence interval is. Furthermore, there is a high variability with the coefficients; most deviate from the 95% confidence interval of the mean value (shaded grey). The test of homogeneity (Q test) was statistically significant (Q(44) = 409.99, p<.05), i.e., the study-to-study variation of the inter-rater reliabilities was considerably higher than would be expected on the basis of random sampling (fixed effects model). To explain the study-to-study variation of correlation coefficients by covariates, meta-regression analyses were calculated.

### Meta-Regression Analyses

Table 2 provides a description of the covariates included in the meta-regression analyses. Table 3 shows the results of the multilevel meta-analyses. These analyses are based on those studies that reported an ICC or r (*n* = 44). For studies with a Kappa coefficient that were included in this study (*n* = 26), no analyses could be performed due to the lack of a statistical approach for carrying out a meta-regression analysis and the comparatively small number of studies.

**Table 2:** Description of the covariates included in the meta-regression analyses (n = 32 studies with 44 coefficients).

Variable (metric)	Range	Mean	Standard Deviation
Number of manuscripts	15→1983	321.98	398.13
Variable (categorical)	Range	Frequency	Percent
*Method*:			
ICC	0–1	35	80
r (RC)	0–1	9	20
*Discipline*:			
Economics/Law	0–1	3	7
Natural Sciences	0–1	7	16
Medical Sciences	0–1	11	25
Social Sciences (RC)	0–1	23	52
*Object of appraisal*:			
Paper	0–1	29	66
Abstract (RC)	0–1	15	34
*Cohort*:			
1950–1979	0–1	15	34
1980–1989	0–1	9	21
1990–1999	0–1	5	11
2000–2008	0–1	8	18
Unknown (RC)	0–1	7	16
*Blinding*:			
Single	0–1	22	50
Double	0–1	3	7
Unknown (RC)	0–1	19	43
*Rating System*:*			
Categorical	0–1	35	80
Metric	0–1	5	11
Unknown (RC)	0–1	4	9

**Note:** RC = reference category in meta-regression analysis. Unknown = this information is missing in a study.

*Rating systems are classified as categorical, if they have nine or fewer categories; in case of more than nine categories, the classification is made as metric [110].

**Table 3:** Multilevel meta-analyses of the metric inter-rater-reliabilities (Fisher-Z √r_tt_ or r).

Model	Model 0Intercept	Model 1Number of Manuscripts	Model 2Method	Model 3Discipline	Model 4Object of Appraisal	Model 5Cohort	Model 6Blinding	Model 7Rating System	Model 8Number of Manuscripts, Rating System
*Fixed effects*	*Coeff / SE*	*Coeff / SE*	*Coeff / SE*	*Coeff / SE*	*Coeff / SE*	*Coeff / SE*	*Coeff / SE*	*Coeff / SE*	*Coeff / SE*
Intercept	.67 / .04*	.77 / .04*	.69 / .07*	.66 / .05*	.74 / .07*	.60 / .08*	.71 / .05*	1.03 / .13*	1.06 / .11*
Number of Manuscripts/100		−.03 / .007*							−.03 / .006*
Method (RC = r)									
ICC			−.02 / .08						
Discipline (RC = Social Sciences)									
Economics/Law				.08 / .13					
Natural Sciences				−.02 / .09					
Medical Sciences				−.006 / .09					
Object of Appraisal (RC = Abstract)									
Paper					−.06 / 0.08				
Cohort (RC = unknown)									
1950–1979						.10 / .09			
1980–1989						.07 / .11			
1990–1999						.15 / .15			
2000–2008						−.007 / .12			
Blinding (RC = unknown)									
Double							.15 / .11		
Single							−.05 / .08		
Rating System (RC = unknown)									
Categorical								−.40 / .14*	−.32 / .11*
Metric								−.33 / .16*	−.33 / .13*
*Random effects* (σ^2^)									
Study Level 3	.03 /.01*	.016 / .009*	.03 / .012*	.03 / .01*	.027 / .01*	.03 / .01*	.03 / .01*	.017 / .01	.0036 / .02
Coefficient Level 2	.01 /.009	.007 / .007	.009 / .009	.009 / .009	.009 / .009	.007 / .008	.005 / .007	.01 /.01	.01 / .02
−2LL	−8.4	−23.7	−8.5	−8.8	−12.7	−10.7	−10.7	−15.3	−30.0
BIC	2.0	−9.9	1.9	8.5	−2.4	10.1	3.2	−1.4	−12.6

**Note**: For each categorical variable, one category was chosen as a reference category (RC, e.g., RC = Social Sciences for the categorical variable discipline). For categorical variables, effect for each predictor variable (a dummy variable representing one of the categories) is a regression coefficient (Coeff) that should be interpreted in relation to its standard error (SE) and the effect of the reference category. Variance components for level 1 are derived from the data, but variance components at level 2 and level 3 indicate the amount of variance that can be explained by differences between studies (level 3) and differences between single reliability coefficients nested within studies (level 2). The loglikelihood test provided by SAS/proc mixed (−2LL) can be used to compare different models, as can also the Bayes Information Criteria (BIC). The smaller the BIC, the better the model is.

*p<.05.

We carried out a series of meta-regression analyses in which we explored the effects of each covariate in isolation and in combination with other covariates. The focus was particularly on tests of the a priori predictions about the effects of the covariates (e.g., publication bias). As Table 3 shows, a total of 9 different models were calculated: Model 0 is the null model. In models 1 to 7 the meta-regression of an IRR on a covariate was determined. In model 8 those covariates were included that emerged as statistically significant in models 1 to 7.

The loglikelihood test provided by SAS/proc mixed (−2LL) can be used to compare different models, as can also the Bayes Information Criteria (BIC). The smaller the BIC, the better the model is. By comparison to the null model, only models 1, 7, and 8 exhibited significant differences in the loglikelihood and BIC, with statistically significant regression coefficients. The covariates method, discipline, object of appraisal, cohort, and blinding were accordingly not significantly correlated to the study-to-study variation (see models 2, 3, 4, 5, and 6).

The statistically significant regression coefficient of −.03 in model 1 can be interpreted as follows: The more manuscripts (divided by 100) that a study is based on, the smaller the reported reliability coefficients are. If the number increases, for instance from 100 manuscripts to 500, the reliability decreases from .40 to .34. By including this covariate, the study-to-study random effects variance declined from .03 (model 0) to .016 (model 1), i.e., 46.6% of the variance between the studies could be explained by the number of manuscripts. This result indicated a distinctly marked publication bias in the case of publication of studies for reliability of peer review. Even when the statistical significance level was adjusted by Bonferroni correction (α divided by the number of single tests), the regression parameter remained statistically significant. There is much evidence in the meta-analysis literature that studies that report relatively high correlations or effect sizes are more likely to be published than results of studies that report low correlations or effect sizes [89]. It seems that low correlations or effect sizes are only published by journals if the results are justified by a huge sample size; high correlations or effect sizes are published even if the sample size of the study is small. The negative correlation found in our meta-analysis between sample size of manuscripts and reliability coefficient confirms this publication bias hypothesis.

A further significant covariate is represented by the rating system. Even, if the statistical significance level is adjusted by Bonferroni correction, the regression parameter of the categorical rating remains statistically significant. It was decisive whether the rating system was reported in a study or not. If the information was conveyed, then this was associated with smaller reliability coefficients (regression coefficients in Table 3: −.40, −.33) than if the information was not conveyed. By considering this covariate, the study-to-study random effects variance decreased from .03 (model 0) to .017 (model 7), i.e., 43.3% of the variance between the studies could be explained. As it can be assumed based on Cicchetti [5] that the mentioning or non-mentioning of information about the rating system provides information about the quality of a study (see above), the IRR about which the individual studies report will vary accordingly with the quality of the studies.

When the statistically significant covariates in models 1 and 7 – number of manuscripts and rating system – were included in a multiple meta-regression analysis, the study-to-study variance fell from 0.03 (model 0) to 0.0036 (model 8), i.e., 86.6% of the variance could be explained with both variables. As the variance component was no longer statistically significant in this model, an approximate homogeneity of the intra-class correlations was present, i.e., the residuals of the meta-regression analysis almost only varied due to sampling error (the desired final result of a meta-analysis).

## Discussion

Meta-analysis tests the replicability and generalizability of results – the hallmark of good science. In this study we present the first meta-analysis for reliability of journal peer reviews. The results of our analyses confirmed the findings of narrative reviews: a low level of IRR: .34 for ICC and r (random effects model) and .17 for Cohen's Kappa. Even when we used different models for calculating the meta-analyses, we arrived at similar results. With respect to Cohen's Kappa, a meta-analysis of studies examining the IRR of the standard practice of peer assessments of quality of care published by Goldman [90] found a similar result: The weighted mean Kappa of 21 independent findings from 13 studies was .31. Based on this result, Goldman [90] considered the IRR of peer assessments to be quite limited and in need of improvement. Neff and Olden [91] concluded in a study on peer review that there are considerable benefits to employing three or four reviewers instead of just two, to minimize decision errors over manuscripts. Marsh, Jayasinghe, and Bond [11] and Jayasinghe, Marsh, and Bond [92] proposed a reader trial approach to peer review to increase IRR: A small number of expert readers are chosen on the basis of research expertise in a certain subdiscipline of a subject. The level of expertise of these readers should be higher than the broader cross-section reviewers in the traditional review system. “The same reader reviewed all the proposal in their subdisciplinary area, rated the quality of both the proposal and the researcher (or team of researcher), provided written comments, and were paid a small emolument” [92] (p. 597). Marsh, Jayasinghe, and Bond [11] found that single-rater reliabilities were much higher for the reader system than for the traditional review approach: For 4.3 readers on average per proposal the IRR of the researcher ratings reaches an acceptable value of .88 for the reader system. Although a high level of IRR is generally seen as desirable, when it comes to peer review some researchers, such as Bailar [93], view agreement as detrimental to the review process: “Too much agreement is in fact a sign that the review process is *not* working well, that reviewers are not properly selected for diversity, and that some are redundant” (p. 138). Although selecting reviewers according to the principle of complementarity (for example, choosing a generalist and a specialist) will lower IRR, the validity of the process can gain, according to Langfeldt [94]: “Low inter-reviewer agreement on a peer panel is no indication of low validity or low legitimacy of the assessments. In fact, it may indicate that the panel is highly competent because it represents a wide sample of the various views on what is good and valuable research” (p. 821).

To explain the study-to-study variation for the inter-rater reliabilities, we calculated meta-regression analyses regarding the metric reliability coefficients. It emerged that neither the type of blinding nor the discipline corresponded to the level of the IRR. With double-blinding, which is already used by many journals as a measure against biases in refereeing [95], an effect at the level of the reviewer agreement can thus be excluded according to our results. This result may point out that such blinding is difficult to accomplish and that reviewers could identify the authors in approximately a quarter to a third of the manuscripts [96]. In each text, there are clues as to the author (e.g., self-citation), and in many cases long-standing researchers in a particular field recognize the author based on these clues [97], [98], [99]. Falagas, Zouglakis, and Kavvadia [100] show that “half the abstracts we reviewed provided information about the origin of the study, despite the fact that instructions to the authors for the preparation of abstracts informed authors that the submissions would undergo masked peer review.”As we mentioned in the section “Material and Methods” with regard to discipline-specific reliabilities, it has been suggested that peer review in the natural and physical sciences should be more reliable because of shared theoretical perspectives. This is in contrast to the social sciences and humanities. In fact, we did not find any effect of discipline, which contradicts the “theoretical paradigms” hypothesis. Our results are in accordance with Cole's statement [101] that a low level of agreement among reviewers reflects the lack of consensus that is prevalent in all scientific disciplines at the ‘research frontier.’ Cole [101] says that usually no one reliably assesses scientific work occurring at the frontiers of research.

Two covariates emerged in the analyses as significant, to achieve approximate homogeneity of the intra-class correlations. On the one hand, the number of manuscripts on which a study is based was statistically significant. We therefore assume a distinctly more marked publication bias for studies on IRR: With a small sample, the results are published only if the reported reliability coefficients are high. If the reported reliability coefficients are low, on the other hand, a study has to be based on a large number of manuscripts to justify publication. Figure 1 also shows this correlation distinctly: The larger the confidence interval of a reliability coefficient, the higher the coefficient will be. This results from the fact that high reliability coefficients are reported more probably by studies with small sample sizes, which are associated with large standard errors and confidence intervals of the estimates.

Apart from the number of manuscripts upon which a study is based, the covariate rating system was also statistically significant. Studies that do not provide information on the rating system report higher IRR coefficients than studies that provide detailed information on the rating system. Failure to mention the rating system must be viewed as an indication of low quality of a study.

The main conclusion of our meta-analysis is that studies that report a high level of IRR are to be considered less credible than those with a low level of IRR. The reason is that high IRR coefficients are mostly based on small sample sizes than low IRR coefficients, which are based mostly on huge sample sizes. In contrast to narrative literature reviews, quantitative meta-analysis weights the study results according to the standard error to get unbiased estimates of the mean IRR. Therefore, meta-analysis should be preferred over narrative reviews. However, future primary studies on IRR of peer reviews that could be included in later meta-analyses should be based on large sample sizes and describe the evaluation sheet/rating system for reviewers in detail.

Very few studies have investigated reviewer agreement with the purpose of identifying the actual reasons behind reviewer disagreement, e.g., by carrying out comparative content analyses of reviewers' comment sheets [102], [103]. For example, LaFollette [104] noted the scarcity of research studies on questions such as how reviewers apply standards and the specific criteria established for making a decision on a manuscript. In-depth studies that address these issues might prove to be fruitful avenues for future investigation [7]. This research should dedicate itself primarily to the dislocational component in the judgment of reviewers as well as differences in strictness or leniency in reviewer's judgments [105], [106].

Studies included in the meta-analyses are marked with an asterisk.
